# A Novel Amino Acid Substitution, Fibrinogen Bβp.Pro234Leu, Associated with Hypofibrinogenemia Causing Impairment of Fibrinogen Assembly and Secretion

**DOI:** 10.3390/ijms21249422

**Published:** 2020-12-10

**Authors:** Takahiro Kaido, Masahiro Yoda, Tomu Kamijo, Shinpei Arai, Chiaki Taira, Yumiko Higuchi, Nobuo Okumura

**Affiliations:** 1 Department of Clinical Laboratory Investigation, Graduate School of Medicine, Shinshu University, Matsumoto 390-8621, Japan; 19ms108h@shinshu-u.ac.jp (T.K.); 19ms113d@shinshu-u.ac.jp (M.Y.); arais@shinshu-u.ac.jp (S.A.); tairacha@shinshu-u.ac.jp (C.T.); sasa0922@shinshu-u.ac.jp (Y.H.); 2 Department of Medical Sciences, Graduate School of Medicine, Science and Technology, Shinshu University, Matsumoto 390-8621, Japan; kamtom@shinshu-u.ac.jp; 3 Department of Laboratory Medicine, Shinshu University Hospital, Matsumoto 390-8621, Japan; 4 Laboratory of Clinical Chemistry and Immunology, Department of Biomedical Laboratory Sciences, School of Health Sciences, Shinshu University, 3-1-1 Asahi, Matsumoto 390-8621, Japan

**Keywords:** congenital fibrinogen disorders, hypofibrinogenemia, fibrinogen Bβ chain, fibrinogen Bβγ complex, multiple amino acid sequence alignment

## Abstract

We identified a novel heterozygous variant, Bβp.Pro234Leu (fibrinogen Tokorozawa), which was suspected to be associated with hypofibrinogenemia. Therefore, we analyzed the assembly and secretion of this fibrinogen using Chinese hamster ovary (CHO) cells. To determine the impact on the synthesis and secretion of fibrinogen of the Bβp.P234L and γp.G242E substitutions, we established recombinant variant fibrinogen-producing CHO cell lines. Synthesis and secretion analyses were performed using an enzyme-linked immunosorbent assay (ELISA) and immunoblotting analysis with the established cell lines. In addition, we performed fibrin polymerization using purified plasma fibrinogen and in-silico analysis. Both Bβp.P234L and γp.G242E impaired the secretion and synthesis of fibrinogen. Moreover, immunoblotting analysis elucidated the mobility migration of the Bβγ complex in Bβp.P234L. On the other hand, the fibrin polymerization of fibrinogen Tokorozawa was similar to that of normal fibrinogen. In-silico analysis revealed that the Bβp.P234 residue is located in the contact region between the Bβ and γ chains and contacts γp.G242 residue. The present study demonstrated that the Bβp.P234L substitution resulted in hypofibrinogenemia by decreasing the assembly and secretion of fibrinogen. Therefore, there is a possibility that substitutions in the contact region between the Bβ and γ chains impact the assembly and secretion of fibrinogen.

## 1. Introduction

Fibrinogen is a 340 kDa plasma glycoprotein involved in hemostasis by forming fibrin [1]. The human fibrinogen genes, *FGA*, *FGB*, and *FGG*, are clustered on chromosome 4, and these genes translate into polypeptides for the pre-pro-Aα chain (644 residues), pre-pro-Bβ chain (491 residues), and pre-pro-γ chain (437 residues) [1]. After cleaving the signal peptide and removing Aαp.630-644, the polypeptide chains in fibrinogen comprise 610 residues (Aα chain), 461 residues (Bβ chain), and 411 residues (γ chain) [1,2]. Each chain is synthesized in hepatocytes and assembled into the Bβγ complex and Aαγ complex. Then, a three-chain monomer (Aα-Bβ-γ) forms by combining the Aα chain and Bβγ complex or Bβ chain and Aαγ complex, and this monomer is held together by each N-terminal portion into a six-chain dimer (Aα-Bβ-γ)_2_, which is then secreted into the circulation [1,3].

Up to 800 congenital fibrinogen disorders (CFDs) have been listed on the Groupe d’études sur l’hémostase et la thrombose (GEHT) database [4]. CFDs are classified according to functional and immunological fibrinogen levels as quantitative or qualitative disorders [5]. Quantitative disorders are afibrinogenemia and hypofibrinogenemia, which correspond to the complete absence of fibrinogen or decreased immunological fibrinogen levels, respectively [6]. Quantitative disorders due to mutations lead to decreased amounts of fibrinogen by causing defects in the synthesis of the constituent chains or in fibrinogen assembly, stability, or secretion [7]. Generally, afibrinogenemia is caused by homozygous or compound mutations, and hypofibrinogenemia is induced by heterozygous mutations [7]. On the other hand, qualitative disorders comprise dysfibrinogenemia and hypodysfibrinogenemia, which have reduced functional fibrinogen levels or disproportionately decrease the functional and immunological levels, respectively [8]. Dysfibrinogenemia is mostly caused by mutations at important sites for the coagulation function [8].

In this study, we identified a novel amino acid substitution associated with hypofibrinogenemia. Furthermore, we analyzed the impact of this substitution on the assembly and secretion of fibrinogen using a Chinese hamster ovary (CHO) cell line.

## 2. Results

### 2.1. Patient Description

The proposita of fibrinogen Tokorozawa was a 36-year-old Japanese female subjected to surgery due to miscarriage at 12 weeks of gestation. The laboratory data before the operation showed that her functional fibrinogen level was 1.36 g/L, and her prothrombin time-international normalized ratio was 1.13. However, fibrin/fibrinogen degradation products, D-dimer, factor VII, factor XIII, and the activated partial thromboplastin time (APTT) were within normal ranges. During the operation, excessive bleeding occurred and 3 g of fibrinogen concentration was administered. After that, she improved. Although she and her family members had not experienced any episodes of abnormal bleeding or thrombosis, she was referred to Shinshu University Hospital to investigate the cause of lower fibrinogen concentration.

### 2.2. Coagulation Screening Tests and DNA Sequence Analysis

For the Tokorozawa proposita, the functional and immunological plasma fibrinogen concentrations (reference range: 1.80–3.50 g/L) were 1.24 and 1.16 g/L, respectively, and the functional/immunological ratio was 1.07 (0.90–1.10). The prothrombin time (PT) and APTT were 11.1 s (10.8–13.2 s) and 28.9 s (23.0–38.0 s), respectively. This proposita was diagnosed with suspected hypofibrinogenemia.

**Figure 1:**
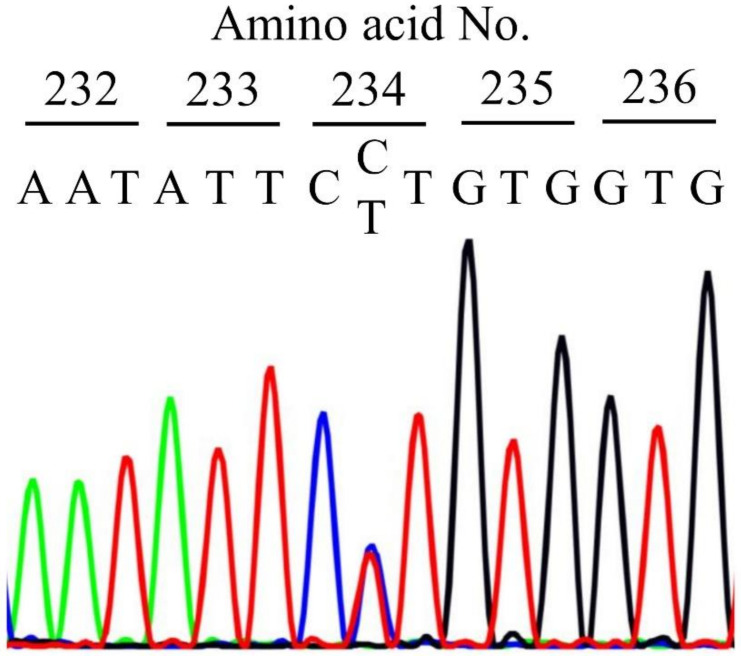
DNA sequence of proposita. The DNA sequence around the mutation position in *FGB* exon 4 is shown. A heterozygous variant c.701C>T was identified (Bβp.P234L). Green: adenine (A), red: thymine (T), black: guanine (G), blue: cytosine (C).

The DNA sequence analysis revealed a heterozygous variant, *FGB* exon 4 c.701C>T, resulting in the substitution of Leu (CTT) for Pro (CCT) at the Bβ204 residue (mature protein: BβP204L) or Bβp.234 residue (native protein: Bβp.P234L) (Figure 1). Any other mutations were not found in *FGA*, *FGB*, and *FGG*. This Bβp.P234L fibrinogen is the first reported case of this mutation worldwide.

### 2.3. Secretion and Synthesis of Variant Fibrinogen in CHO Cells

We established a Bβp.P234L fibrinogen-producing CHO cell line and a γp.G242E fibrinogen-producing CHO cell line, since the Bβp.P234 residue contacts the γp.G242 residue. We will explain the contact between Bβp.P234 and γp.G242 residues in detail in a later section.

**Figure 2:**
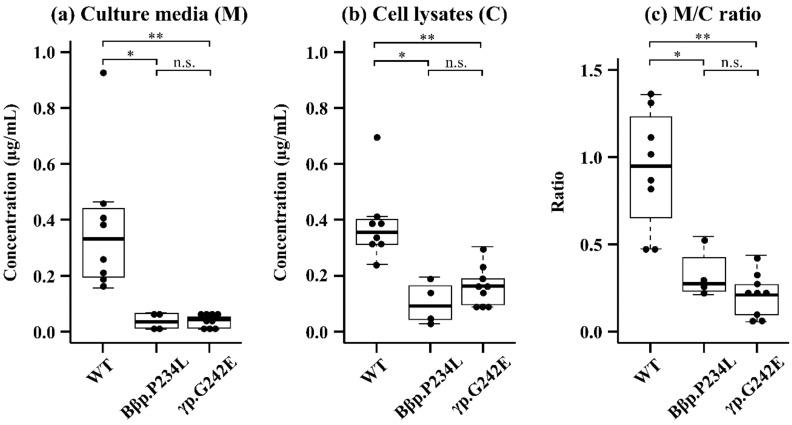
Secretion and synthesis of fibrinogen in Chinese hamster ovary (CHO) cells. Fibrinogen concentrations in the culture media (Panel (**a**)) and cell lysates (Panel (**b**)) were measured using ELISA. Panel (**c**) shows the ratio in the medium to the cell lysate. Box plots and the central bar show the interquartile range and median, respectively. The whiskers indicate the minimum and maximum value excluding outliers. Dots represent individual values. Concentrations were assessed for clones expressing wild-type (WT, *n* = 8), Bβp.P234L (*n* = 4), and γp.G242E (*n* = 9). The significance of differences among WT and variant fibrinogen-producing CHO cells was assessed using the Kruskal-Walls test and the Steel-Dwass test. * *p* < 0.05; ** *p* < 0.01; n.s.: Non-significant.

The fibrinogen concentrations of wild-type (WT) (*n* = 8, median (interquartile range)) were 0.33 (0.20–0.43) μg/mL in the culture media and 0.36 (0.31–0.39) μg/mL in cell lysates, resulting in a ratio of culture media to cell lysates (M/C ratio) of 0.95 (0.73–1.18). In the culture media of the variant fibrinogen-producing CHO cells, the fibrinogen concentrations of Bβp.P234L (*n* = 4, 0.04 (0.01–0.06) μg/mL) and γp.G242E (*n* = 9, 0.04 (0.01–0.05) μg/mL) were significantly lower than that of WT (Figure 2a). In the cell lysates, fibrinogen concentrations of Bβp.P234L (0.09 (0.05–0.14) μg/mL) and γp.G242E (0.16 (0.09–0.19) μg/mL) were also significantly lower (Figure 2b). In addition, the M/C ratio of variant fibrinogen-producing CHO cells (Bβp.P234L: 0.27 (0.24–0.36), γp.G242E: 0.21 (0.09–0.27)) was significantly lower than that of WT (Figure 2c). There were no significant differences in fibrinogen production between the two variant fibrinogen-producing CHO cell lines.

**Figure 3:**
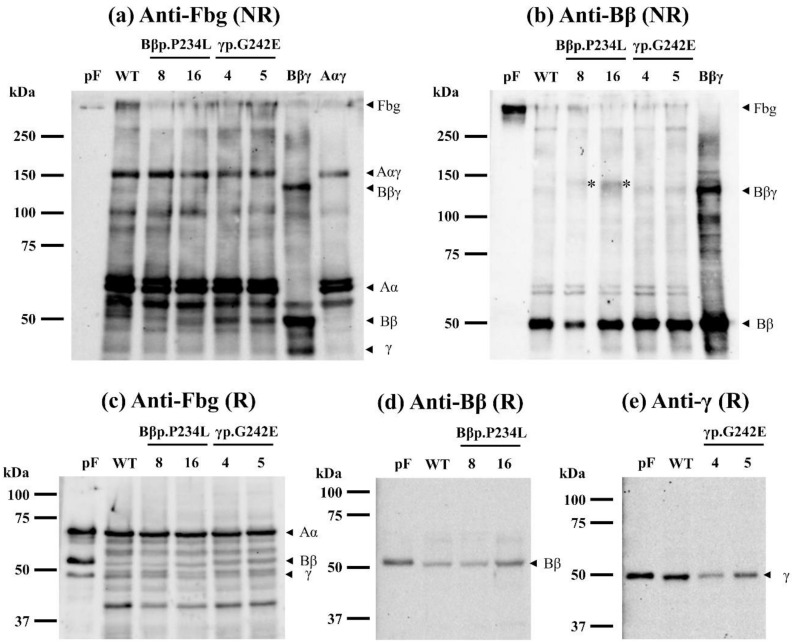
Immunoblotting analysis of CHO cell lysates. Panels (**a**,**b**) were produced in non-reducing conditions using 7.5% gels, and Panels (**c**–**e**) were produced in reducing conditions using 10% gels. An anti-human fibrinogen polyclonal antibody (Panel (**a**,**c**)), an anti-human fibrinogen Bβ chain polyclonal antibody (Panel (**b**,**d**)), and an anti-human fibrinogen γ chain monoclonal antibody (Panel (**e**)) were used. A size marker is shown on the left side of each panel. Bands shown with an asterisk ‘*’ in Panel (**b**) indicate the migrated Bβγ complex. pF: Purified recombinant wild-type fibrinogen; WT: Wild-type fibrinogen; Bβp.P234L: Recombinant Bβp.P234L fibrinogen; γp.G242E: Recombinant γp.G242E fibrinogen; Bβγ: Wild-type Bβ and γ chains; Aαγ: Wild-type Aα chain and γ chain-producing CHO cell lysate. The number with each variant fibrinogen shows the clone number. NR: Non-reducing conditions; R: Reducing conditions; Fbg: Fibrinogen.

The immunoblotting analysis demonstrated that the variant Bβ chain and variant γ chain were synthesized and variant fibrinogen was slightly assembled in each variant fibrinogen-producing CHO cells (Figure 3a,c–e). In addition, we performed the immunoblotting analysis in a non-reducing condition using an anti-fibrinogen Bβ chain polyclonal antibody. The mobility of the Bβγ band in γp.G242E fibrinogen-producing cells was similar to that in WT fibrinogen-producing cells, whereas the mobility of the Bβγ band in Bβp.P234L fibrinogen-producing cells was lower than that in WT fibrinogen-producing cells (shown with an asterisk in Figure 3b). We speculated that the tertiary structure of the Bβγ complex comprised of Bβp.P234L is aberrant and leads to a mobility shift in non-reducing conditions. On the other hand, the band of the Aαγ complex had similar mobility in all fibrinogens, as shown in Figure 3a. We did not analyze the mRNA expression levels of variant Bβ or γ chains, since these mutant proteins were synthesized clearly in CHO cells (see Figure 3d,e).

### 2.4. Characterization of Purified Plasma Fibrinogens and Thrombin-Catalyzed Fibrin Polymerization

**Figure 4:**
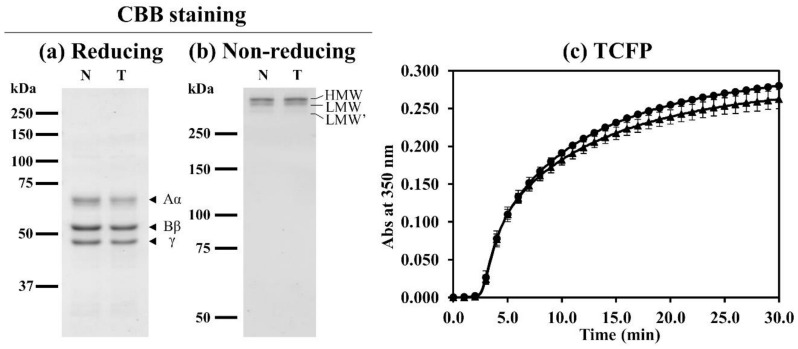
Characterization of purified plasma fibrinogens and thrombin-catalyzed fibrin polymerization. Purified plasma fibrinogens were resolved using SDS-PAGE in reducing conditions (Panel (**a**)) or non-reducing conditions (Panels (**b**)) and stained with Coomassie brilliant blue R-250 (CBB). A size marker is shown on the left side of each panel. N: Normal fibrinogen; T: Fibrinogen Tokorozawa; HMW: High molecular weight-fibrinogen; LMW: Low molecular weight-fibrinogen; LMW’: LMW’-fibrinogen. Panel (**c**): Fibrin polymerization reactions were performed in the following conditions; thrombin: 0.05 U/mL, fibrinogen: 0.18 mg/mL, CaCl_2_: 1 mM. Data are presented as the mean ± SD. Normal fibrinogen (closed circle), fibrinogen Tokorozawa (closed triangle). TCFP: Thrombin-catalyzed fibrin polymerization.

Purified plasma fibrinogens (normal control and fibrinogen Tokorozawa) showed the typical pattern for Aα, Bβ, and γ chains on sodium dodecyl sulfate-polyacrylamide gel electrophoresis (SDS-PAGE) in reducing conditions. In addition, three fibrinogen bands (high molecular weight-fibrinogen (HMW-fibrinogen; 340 kDa), low molecular weight-fibrinogen (LMW-fibrinogen; 305 kDa), or LMW’-fibrinogen (270 kDa) were identified in non-reducing conditions (Figure 4a,b). The structural difference between LMW-fibrinogen and LMW’-fibrinogen was the removal of a 35 kDa carboxyterminal polypeptide from one or two Aα chain(s), respectively [9,10].

We performed thrombin-catalyzed fibrin polymerization at room temperature using purified plasma fibrinogen, and turbidity curves were obtained as shown in Figure 4c. Moreover, three parameters; lag time, maximum-slope (Vmax), and absorbance-change at 30 min (ΔAbs_30min_) were obtained from the turbidity curves. The polymerization curve of fibrinogen Tokorozawa (heterozygous Bβp.P234L) was similar to that of normal fibrinogen. Moreover, there were no significant differences between normal fibrinogen and fibrinogen Tokorozawa in either lag time (normal fibrinogen (mean ± SD): 2.4 ± 0.20 min vs. fibrinogen Tokorozawa: 2.5 ± 0.10 min), Vmax (8.9 ± 0.29 × 10^−4^/s vs. 9.1 ± 0.59 × 10^−4^/s), or ΔAbs_30min_ (0.280 ± 0.002 vs. 0.262 ± 0.013). Therefore, the coagulation function of fibrinogen Tokorozawa was normal.

### 2.5. Identification of Contact Residues and Multiple Amino Acid Sequence Alignment of the Mutation Site

**Figure 5:**
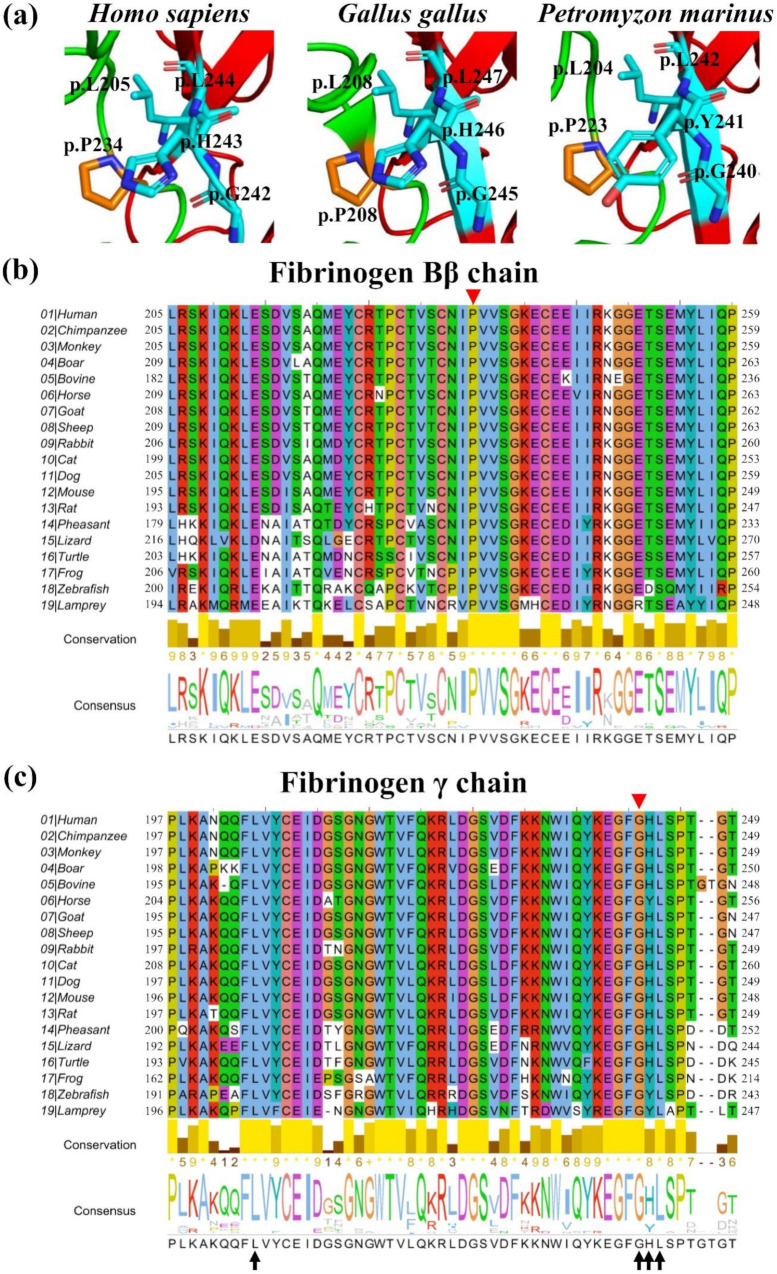
The structure of the Bβ and γ chain contact region and multiple amino acid sequence alignment. Panel (**a**) shows the Bβ and γ chain contact region of *Homo sapiens* fibrinogen (PDB ID: 2FFD), *Gallus gallus* fibrinogen (PDB ID: 1M1J), and *Petromyzon marinus* fibrinogen (PDB ID: 1LWU). The residues corresponding to the human fibrinogen Bβp.P234 residue and human fibrinogen γ chain residues (γp.L205, γp.G242, γp.H243, and γp.L244) are indicated in orange and cyan, respectively. Green and red show the Bβ and γ chains, respectively. Multiple amino acid sequence alignments of the Bβ or γ chain are shown in Panels (**b**,**c**), respectively. The sequence alignment was performed using the Clustal Omega program [11] and rendered with Jalview [12]. We referenced each animal fibrinogen amino acid sequence in UniProt [13]. Residues in the alignment are colored in accordance with the ClustalX [14] shading model: Color is only applied when that residue’s abundance in the column is above a residue-specific threshold, highlighting potentially important residues or patterns of conservation. The raw of conservation indicates the conservation of the physicochemical properties and shows that the lighter the yellow or the higher the value, the more conserved in the column. Asterisks ‘*’ indicate what is absolutely conserved in a column, plus ‘+’ indicates columns where physicochemical properties are conserved in the column with a score of 10. The raw consensus indicates the percentage of residues in the column, and the consensus sequence is shown at the bottom. The residues corresponding to mutation positions (human fibrinogen Bβp.P234L and γp.G242E) are shown with red arrowheads. Arrows in Panel (**c**) show the contact residues with the human fibrinogen Bβp.P234 residue.

The Bβp.P234 residue is located in the surface area of the Bβ chain near the γ chain. To reveal the residues in the γ chain that contact the Bβp.P234 residue, we analyzed these residues using the script and four crystal structures of human fibrinogen (PDB ID: 2FFD, 1LTJ, 1FZA, 3GHG). From these structures, the residues that contact the Bβp.P234 residue were γp.G242, γp.H243, and γp.L244. In addition, for 2FFD and 1LTJ, the γp.L205 residue also contacted Bβp.P234 (Figure 5a). According to the human fibrinogen database, the substitution at γp.G242 (γp.G242E) was the only one reported among these residues [4]. In addition, the structural analysis of *Gallus gallus* fibrinogen and *Petromyzon marinus* fibrinogen revealed that the former fibrinogen Bβp.P208 and latter fibrinogen Bβp.P223 (these residues correspond to human fibrinogen Bβp.P234) also contact the residues corresponding to human fibrinogen γp.L205, γp.G242, γp.H243, and γp.L244 (*G. gallus*: γp.L208 γp.G245, γp.H246, and γp.L247, *P. marinus*: γp.L204, γp.G240, γp.Y241, and γp.L242) (Figure 5a).

To determine the highly conserved residues, we performed the multiple amino acid sequence alignment. As shown in Figure 5b,c, the residue corresponding to the human fibrinogen Bβp.P234 residue was conserved in all 19 species. Moreover, the residue corresponding to the human γp.G242 residue was also conserved. The concordance percentage with human fibrinogen was 100% for the corresponding residues with human fibrinogen γp.L205, γp.G242, and γp.L244 and 77.8% (14/18 species except human) for the corresponding residues to human fibrinogen γp.H243. Horse, frog, zebrafish, and lamprey fibrinogen have tyrosine but not histidine among the residues corresponding to human fibrinogen γp.H243. However, these residues are aromatic residues, and this position exhibits high levels of conservation. Therefore, the related residues corresponding to human fibrinogen Bβp.P234 are highly conserved in many species.

### 2.6. In-Silico Molecular Analysis

**Figure 6:**
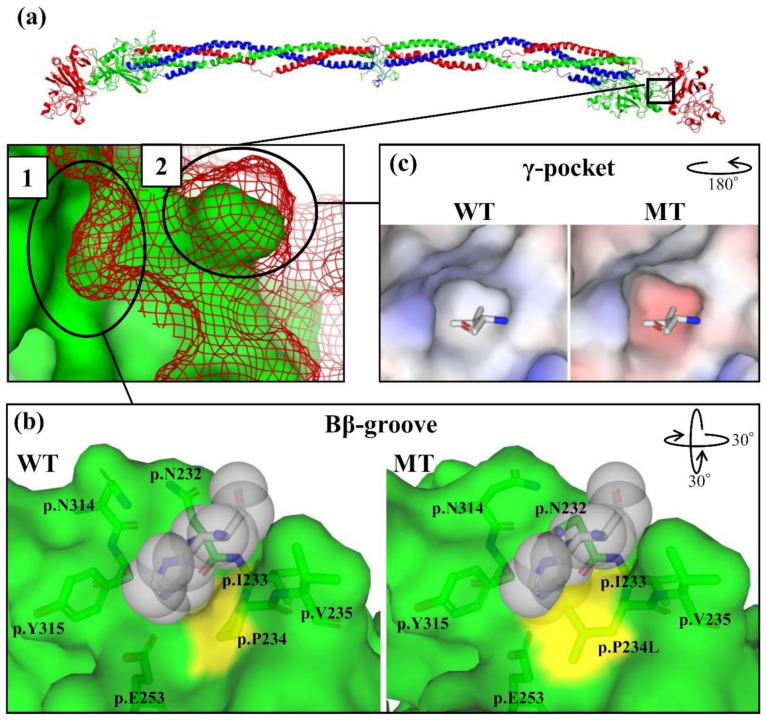
The impacts on each chain structure of mutations. Panel (**a**) (upper side) shows the whole fibrinogen structure (PDB ID: 3GHG) (Aα chain: Blue, Bβ chain: Green, γ chain: Red). Note that this structure lacks Aαp.20-45, Aαp.220-629, Bβp.31-87, Bβp.489-491, γp.27-39, and γp.422-437. Panel (**a**) (lower side) shows the Bβγ chain contact region indicated with a square in the upper panel. The green surface and red mesh indicate the surface area of the Bβ and γ chains, respectively. Panel (**b**) shows the surface structure around region 1. A gray stick residue shown with a space-filling model is γp.H243, and this residue is in the Bβ-groove. The component residue of this groove is shown using a green stick. The yellow region indicates the Bβp.P234L mutation position. Panel (**c**) shows region 2 at another angle. The stick residue is Bβp.V235, and this residue is in the γ-pocket. The γ chain is indicated using the electrostatic potential (blue and red indicate more positive potential and more negative potential, respectively). The rotated view compared with the lower Panel (**a**) according to the angle guide in the upper right of each panel. WT: Wild-type; MT: Mutant-type.

To reveal the impact on the Bβγ complex structure from each mutation, we performed the in-silico molecular analysis, and these results are shown in Figure 6 and details of the region are shown in Figure 6b,c. The contact region between the Bβ and γ chains is shown in Figure 6a. The groove that the γp.H243 residue enters into is composed of Bβp.N232, Bβp.I233, Bβp.P234, Bβp.V235, Bβp.E253, Bβp.N314, and Bβp.Y315 (Figure 6b). We call the groove the “Bβ-groove” in this study. In the mutant-type (MT) with the Bβp.P234L substitution, the Bβ-groove was swelled by this substitution and clashed with the γp.H243 residue. Moreover, both the side chain angles of the Bβp.N232 and Bβp.N314 residues were changed for fixing the static hindrance by the Bβp.P234L substitution. On the other hand, the γp.G242 residue is the component of the pocket, which the Bβp.V235 residue enters into (Figure 6c). This pocket was composed of γp.L205, γp.G240, γp.F241, γp.G242, γp.H243, γp.L244, γp.F252, γp.W253, γp.L254, and γp.K258. We call the pocket the “γ-pocket” in this study. In the MT of the γp.G242E substitution, the shape of the γ-pocket was similar to that of WT, but its electrostatic potential changed to negative from neutral.

## 3. Discussion

We identified the novel heterozygous variant, Bβp.P234L, which was designated as fibrinogen Tokorozawa. We considered that this substitution caused hypofibrinogenemia since the proposita’s immunological plasma fibrinogen concentration was low but the functional/immunological ratio was normal. Therefore, we performed thrombin-catalyzed fibrin polymerization using purified plasma fibrinogen and analyzed the secretion and synthesis of variant fibrinogen using CHO cells. The results of thrombin-catalyzed fibrin polymerization revealed that the coagulation function of fibrinogen Tokorozawa was normal. Moreover, in the analysis of secretion and synthesis, although the variant Bβ chain was synthesized normally in the cell, a small amount of Bβp.P234L fibrinogen was assembled in the cell, and just a limited proportion was secreted into the culture medium. In other words, the Bβp.P234L substitution impaired both the assembly and secretion of fibrinogen. Therefore, we demonstrated that the Bβp.P234L substitution caused hypofibrinogenemia.

In addition, since the Bβp.P234 residue was in contact with the γp.G242 residue, we established a recombinant γp.G242E fibrinogen-producing CHO cell line and also analyzed the secretion and synthesis of fibrinogen. Two cases of γp.G242E fibrinogen have been reported as fibrinogen Caracas and fibrinogen French Basque, and this substitution was also associated with hypofibrinogenemia [15,16]. Moreover, Marchi et al. demonstrated the absence of the variant fibrinogen in the Caracas patient plasma using the electrospray time of flight-mass spectrometry [15]. Our findings showed that the secretion from the Bβp.P234L fibrinogen-producing CHO cells was impaired, similar to that from the γp.G242E fibrinogen-producing CHO cells, therefore, we speculated that the Bβp.P234L fibrinogen was also absent in the Tokorozawa patient plasma, since the fibrinogen concentration in the culture media of variant fibrinogen-producing CHO cells was markedly reduced. Furthermore, it is noteworthy that the γp.G242E substitution reduced not only secretion but also the assembly of fibrinogen as with the recombinant Bβp.P234L fibrinogen-producing CHO cells. Therefore, there is a possibility that around the contact region between the Bβp.P234 residue and/or the γp.G242 residue the secretion and assembly of fibrinogen is involved.

The Bβp.P234 residue is located on the Bβ chain surface area where the contact with the γ chain occurs. Moreover, there are interchain disulfide bonds, Aαp.C180-γp.C161, Aαp.C184-Bβp.C223, and Bβp.C227-γp.C165, around the C-terminal side contact region between the Bβ and γ chains [1]. Zhang et al. demonstrated that the disruption of these interchain disulfide bonds allowed the three chains to assemble into a six-chain dimer but only a small amount of fibrinogen was secreted [17]. Therefore, we speculated that it is important for the secretion of fibrinogen to be assembled on the C-terminal side of three chains in the correct form. Since the Bβp.P234L and γp.G242E substitutions were located in the Bβ chain and γ chain contact region, the cause of the decreased secretion may be to impair assembly into the correct form. Moreover, we considered that the cause of the decreased assembly of fibrinogen was mainly due to the impaired Bβγ complex formation. Actually, the Bβγ complex band in the recombinant Bβp.P234L fibrinogen-producing CHO cells was so affected that its mobility was altered. However, the mobility of the Bβγ complex band in recombinant γp.G242E fibrinogen-producing CHO cells was not changed. This result might demonstrate that the Bβp.P234L substitution had a larger impact on the Bβγ complex formation than the γp.G242E substitution. This hypothesis was supported by the in-silico molecular analysis. The Bβp.P234L substitution impacted the Bβ-groove structure, thereby causing static hindrance with γp.H243. In contrast, the γp.G242E substitution had a smaller impact on the structure than the Bβp.P234L substitution since the γp.G242E substitution mainly influenced the electrostatic potential of the γ-pocket, and the electrostatic potential of the Bβp.V235 residue was neutral.

The causative mutations for quantitative disorders in the Bβ and γ chains were clustered in each highly conserved C-terminal globular domain [18]. Therefore, we compared the amino acid sequence of each Bβ and γ chain among animals by the multiple sequence alignment. The Bβp.P234 and γp.G242 residues were highly conserved beyond the species. Moreover, since the fibrinogen structures of *G. gallus* and *P. marinus* have been registered in the PDB data bank among these species except humans, we confirmed that the region of these species’ fibrinogen structure corresponded to the region around the human fibrinogen Bβp.P234 residue. Notably, both residues of *G. gallus* and *P. marinus* corresponding to the human fibrinogen Bβp.P234 residue were contacted with the same residues of human fibrinogen. In addition, these γ chain residues were also highly conserved in various animals. Therefore, it is considered that these residues play an important role in proper assembly into the Bβγ complex and secretion as a six-chain dimer.

## 4. Materials and Methods

This study was approved by the Ethics Review Board of Shinshu University School of Medicine (approval number 603: 5 December 2017). After the informed consent had been obtained from the proposita, blood samples were collected for biochemical and genetic analyses.

### 4.1. Coagulation Screening Tests and DNA Sequencing of the Fibrinogen Gene

Blood collection from the proposita and plasma separation were performed as described previously [19]. PT, APTT, fibrinogen concentrations, which were assessed using the thrombin time method, and immunological fibrinogen concentrations, which were assessed using a latex photometric immunoassay, were measured as described previously [19].

To amplify all exons and exon–intron boundaries in *FGA*, *FGB*, and *FGG*, polymerase chain reaction (PCR) primers were designed as described previously [20]. DNA was amplified using PCR, the PCR products were purified from 1% agarose gels, and purified PCR products were sequenced directly as described previously [19].

### 4.2. Establishment of Variant Fibrinogen-Producing Chinese Hamster Ovary Cells

A Bβp.P234L substitution was introduced into the fibrinogen Bβ chain expression vector pMLP-Bβ plasmid (kindly provided by Lord ST, University of North Carolina, Chapel Hill, NC, USA), which contained WT Bβ cDNA, using a QuikChange II Site-Directed Mutagenesis Kit (Stratagene, La Jolla, CA) and the primers (forward: 5′-CACTGTCAGTTGCAATATTCTTGTGGTGTCTGGC-3′, reverse: 5′-GCCAGACACCACAAGAATATTGCAACTGACAGTG-3′). Mutated plasmids were co-transfected with histidinol selection plasmid into CHO cells that expressed the WT human fibrinogen Aα chain and γ chain, and colonies were selected on histidinol (Aldrich Chem., Milwaukee, WI, USA) [20]. Moreover, the establishment of a recombinant γp.G242E fibrinogen-producing CHO cell line was performed using the fibrinogen γ chain expression vector pMLP-γ plasmid (kindly provided by Lord ST), which contained WT γ cDNA, and had the γp.G242E mutation introduced using these primers (forward: 5′-GAAGGATTTGAACATCTGTCTCCTACTGGCACAACAG-3′, reverse: 5′-CTGTTGTGCCAGTAGGAGACAGATGTTCAAATCCTTC-3′) and CHO cells that expressed the WT human fibrinogen Aα chain and Bβ chain as described above. As a control, we used eight clones of the WT human fibrinogen-producing CHO cell established as described previously [21].

### 4.3. Enzyme-Linked Immunosorbent Assay (ELISA) and Immunoblotting Analysis

Culture media and cell lysates for ELISA were prepared as follows. CHO cells were cultured to confluence in 60-mm dishes (approximately 3.0 × 10^6^ cells) and the conditioned media was harvested 1 day after reaching confluence (6–8 days after seeding). Then, from the same dish, CHO cells were harvested. These were washed three times, and dissolved in a 50 mM Tris-HCl pH 8.0 buffer added with 0.1% IGEPAL CA-630 (Sigma-Aldrich, St Louis, MO, USA) and 10 mM phenylmethylsulfonyl fluoride (Sigma-Aldrich, St Louis, MO, USA). Fibrinogen concentrations in these samples were measured using ELISA as previously described [20].

Cell lysates of WT and variant fibrinogen-producing CHO cells were utilized for immunoblotting analyses. SDS-PAGE and the immunoblotting analysis were performed as described previously [20], and we used a rabbit anti-human fibrinogen polyclonal antibody (DAKO, glostrup, Denmark), a rabbit anti-human fibrinogen Bβ chain polyclonal antibody (Chemicon International, Temecula, CA, USA), and a mouse anti-human fibrinogen γ chain monoclonal antibody (2G10; Accurate Chemical and Scientific, Westbury, NY, USA). The detection of bands was performed using a ChemiDoc XRS Plus (BIO-RAD, CA, USA).

### 4.4. Purification of Plasma Fibrinogen

The purification of plasma fibrinogen from a healthy person and proposita (Tokorozawa) was performed using immunoaffinity chromatography with an IF-1 monoclonal antibody (LSI Medience, Tokyo, Japan)-conjugated Sepharose 4B column, and purified fibrinogen concentrations were determined as described previously [19]. The purity and characterization of each purified fibrinogen was analyzed by SDS-PAGE in non-reducing conditions (7.5% polyacrylamide gel) and reducing conditions (10% polyacrylamide gel) and stained with Coomassie Brilliant Blue R-250.

### 4.5. Thrombin-Catalyzed Fibrin Polymerization

Turbidity curves of fibrin polymerization were recorded at 350 nm using a UV-1280 (Shimadzu, Tokyo, Japan). Recordings were performed as described previously [19]. The final concentration in a 20 mM N-[2-hydroxyethyl] piperazine-N’-[2-ethansulfonic acid] pH 7.4, 0.12 M NaCl buffer was as follows: Human α-thrombin (Enzyme Research Laboratories, South Bend, MA, USA): 0.05 U/mL, fibrinogen: 0.18 mg/mL, and CaCl_2_: 1 mM. Three parameters: Lag time, Vmax, and ΔAbs_30min_, were obtained from the turbidity curves as described previously [19]. Reactions were performed in triplicate experiments for each sample.

### 4.6. Identification of Contact Residues between the Bβ and γ Chains

The human fibrinogen crystal structures were obtained from the protein databank (PDB ID: 2FFD, 1LTJ, 1FZA, and 3GHG) [22,23,24,25]. The interface residues between the mutation position in the Bβ and γ chains were analyzed using the Python script “InterfaceResidues.py” (available at http://www.protein.osaka-u.ac.jp/rcsfp/supracryst/suzuki/jpxtal/Katsutani/InterfaceResidues.py). Moreover, using the fibrinogen structure of pheasant (*G. gallus*, PDB ID: 1M1J [26]) and lamprey (*P. marinus*, PDB ID: 1LWU [27]), the γ chain interface residues contacting with residues corresponding to the human fibrinogen Bβp.P234 residue were analyzed as described above. All figures with molecular modeling were prepared using PyMOL [28].

### 4.7. Multiple Amino Acid Sequence Alignment of the Mutation Site

We performed the multiple amino acid sequence alignment of the fibrinogen Bβ chain or γ chain using the Clustal Omega program [11], and the multiple sequence alignment was rendered with Jalview [12] and residues in the alignment were colored according to the ClustalX [14]. We compared the amino acid sequences of 19 species registered in UniProt [13], including human (*Homo sapiens*, Bβ chain ID: P02675 and γ chain ID: P02679-2), chimpanzee (*Pan troglodytes*, H2QQB4 and H2RDH7), monkey (*Macaca mulatta*, F6UZ87 and F6UZ20), boar (*Sus scrofa*, F1RX37 and F1RX35), bovine (*Bos taurus*, P02676 and P12799), horse (*Equus caballus*, F6PH38 and F6W2Y1), goat (*Capra hircus*, A0A452ENA3 and A0A452EN73), sheep (*Ovis aries*, W5NQ45 and W5Q5A6), rabbit (*Oryctolagus cuniculus*, G1T0W8 and G1TKX3), cat (*Felis catus*, M3WII3 and M3WN28), dog (*Canis lupus familiaris*, F1PGS2 and F1P8G0), mouse (*Mus musculus*, Q8K0E8 and Q8VCM7), rat (*Rattus norvegicus*, P14480 and P02680), pheasant (*G. gallus*, Q02020 and E1BV78), lizard (*Anolis carolinensis*, G1KRF4 and G1KRE0), turtle (*Pelodiscus sinensis*, K7FPL9 and K7FHU9), frog (*Xenopus tropicalis*, B0JZ09 and A0A6I8SV37), zebrafish (*Danio rerio*, Q6NYE1 and Q7ZVG7), and lamprey (*P. marinus*, P02678 and P04115).

### 4.8. In-Silico Molecular Analysis

The fibrinogen structure models with Bβp.P234L or γp.G242E substitutions were prepared as follows. One hundred models of each mutation were prepared using Modeller9.24 [29] and either chain B or chain C of the fibrinogen crystal structure (PDB ID: 2FFD). We selected the model with the lowest molpdf score, which is the Modeller objective function. The contact residues with Bβp.V235 or γp.H243 residues were determined using the Python script as described above. In addition, the electrostatic potential surface map of the γ chain was calculated using the adaptive Poisson–Boltzmann solver Electrostatics of the PyMOL plugin, and all figures of the modeling structure were prepared using PyMOL [28].

### 4.9. Statistical Analysis

The statistical analysis was performed using the EZR software [30]. The Kruskal-Walls test and the Steel-Dwass test were used to compare the fibrinogen production. The Welch’s *t*-test was used to compare the three parameters of thrombin-catalyzed fibrin polymerization. A difference was considered to be significant when the *p*-value was <0.05. Box-and-whisker plots and dot plots were prepared using the R software (version 3.5.1) [31].

## 5. Conclusions

We identified a novel amino acid substitution in the Bβ chain, Bβp.P234L, and designated this as fibrinogen Tokorozawa. This substitution was associated with hypofibrinogenemia by impairing the assembly and secretion of fibrinogen. Moreover, the γp.G242E substitution, which is located in the contact region of the Bβp.P234 residue, also inhibited the assembly and secretion of fibrinogen. Therefore, there is a possibility that the contact region between the Bβ and γ chains has a principal role in the assembly and secretion of fibrinogen.

## References

[1] Weisel J.W., Litvinov R.I. (2017). Fibrin Formation, Structure and Properties. Subcell. Biochem..

[2] Farrell D.H., Huang S., Davie E.W. (1993). Processing of the carboxyl 15-amino acid extension in the alpha-chain of fibrinogen. J. Biol. Chem..

[3] Weisel J.W. (2005). Fibrinogen and fibrin. Adv. Protein Chem..

[4] Human Fibrinogen Database (2020). Groupe D’etude sur L’hemostase et la Thrombose Human Fibrinogen Database Release 50.

[5] Casini A., Undas A., Palla R., Thachil J., de Moerloose P. (2018). Diagnosis and classification of congenital fibrinogen disorders: Communication from the SSC of the ISTH. J. Thromb. Haemost..

[6] Neerman-Arbez M., Casini A. (2018). Clinical Consequences and Molecular Bases of Low Fibrinogen Levels. Int. J. Mol. Sci..

[7] Simurda T., Brunclikova M., Asselta R., Caccia S., Zolkova J., Kolkova Z., Loderer D., Skornova I., Hudecek J., Lasabova Z. (2020). Genetic Variants in the FGB and FGG Genes Mapping in the Beta and Gamma Nodules of the Fibrinogen Molecule in Congenital Quantitative Fibrinogen Disorders Associated with a Thrombotic Phenotype. Int. J. Mol. Sci..

[8] Smith N., Bornikova L., Noetzli L., Guglielmone H., Minoldo S., Backos D.S., Jacobson L., Thornburg C.D., Escobar M., White-Adams T.C. (2018). Identification and characterization of novel mutations implicated in congenital fibrinogen disorders. Res. Pract. Thromb. Haemost..

[9] Holm B., Godal H.C. (1984). Quantitation of the three normally-occurring plasma fibrinogens in health and during so-called “acute phase” by SDS electrophoresis of fibrin obtained from EDTA-plasma. Thromb. Res..

[10] Holm B., Nilsen D.W., Kierulf P., Godal H.C. (1985). Purification and characterization of 3 fibrinogens with different molecular weights obtained from normal human plasma. Thromb. Res..

[11] Sievers F., Wilm A., Dineen D., Gibson T.J., Karplus K., Li W., Lopez R., McWilliam H., Remmert M., Söding J. (2011). Fast, scalable generation of high-quality protein multiple sequence alignments using Clustal Omega. Mol. Syst. Biol..

[12] Waterhouse A.M., Procter J.B., Martin D.M.A., Clamp M., Barton G.J. (2009). Jalview Version 2—A multiple sequence alignment editor and analysis workbench. Bioinformatics.

[13] UniProt Consortium (2019). UniProt: A worldwide hub of protein knowledge. Nucleic Acids Res..

[14] Thompson J.D., Gibson T.J., Higgins D.G. (2002). Multiple sequence alignment using ClustalW and ClustalX. Curr. Protoc. Bioinform..

[15] Marchi R., Brennan S., Mijares M. (2016). A novel mutation in the fibrinogen γ-chain 216 Gly>Glu causes hypofibrinogenemia. Thromb. Res..

[16] Bauduer F., Mimoun A., Ménard F., de Mazancourt P. (2018). The fibrinogen FGG p.Gly242Glu: A rare mutation associated with hypofibrinogenemia. Blood Coagul. Fibrinolysis.

[17] Zhang J.Z., Redman C.M. (1994). Role of interchain disulfide bonds on the assembly and secretion of human fibrinogen. J. Biol. Chem..

[18] Neerman-Arbez M., de Moerloose P., Casini A. (2016). Laboratory and Genetic Investigation of Mutations Accounting for Congenital Fibrinogen Disorders. Semin. Thromb. Hemost..

[19] Kaido T., Yoda M., Kamijo T., Taira C., Higuchi Y., Okumura N. (2020). Comparison of molecular structure and fibrin polymerization between two Bβ-chain N-terminal region fibrinogen variants, Bβp.G45C and Bβp.R74C. Int. J. Hematol..

[20] Terasawa F., Okumura N., Kitano K., Hayashida N., Shimosaka M., Okazaki M., Lord S.T. (1999). Hypofibrinogenemia Associated With a Heterozygous Missense Mutation γ153Cys to Arg (Matsumoto IV): In Vitro Expression Demonstrates Defective Secretion of the Variant Fibrinogen. Blood.

[21] Yoda M., Kaido T., Taira C., Higuchi Y., Arai S., Okumura N. (2020). Congenital fibrinogen disorder with a compound heterozygote possessing two novel FGB mutations, one qualitative and the other quantitative. Thromb. Res..

[22] Betts L., Merenbloom B.K., Lord S.T. (2006). The structure of fibrinogen fragment D with the “A” knob peptide GPRVVE. J. Thromb. Haemost..

[23] Kostelansky M.S., Betts L., Gorkun O.V., Lord S.T. (2002). 2.8 Å Crystal Structures of Recombinant Fibrinogen Fragment D with and without Two Peptide Ligands: GHRP Binding to the “b” Site Disrupts Its Nearby Calcium-binding Site. Biochemistry.

[24] Spraggon G., Everse S.J., Doolittle R.F. (1997). Crystal structures of fragment D from human fibrinogen and its crosslinked counterpart from fibrin. Nature.

[25] Kollman J.M., Pandi L., Sawaya M.R., Riley M., Doolittle R.F. (2009). Crystal structure of human fibrinogen. Biochemistry.

[26] Yang Z., Kollman J.M., Pandi L., Doolittle R.F. (2001). Crystal structure of native chicken fibrinogen at 2.7 A resolution. Biochemistry.

[27] Yang Z., Spraggon G., Pandi L., Everse S.J., Riley M., Doolittle R.F. (2002). Crystal structure of fragment D from lamprey fibrinogen complexed with the peptide Gly-His-Arg-Pro-amide. Biochemistry.

[28] Schrodinger, LLC (2015). The PyMOL Molecular Graphics System, Version 2.0.

[29] Sali A., Blundell T.L. (1993). Comparative protein modelling by satisfaction of spatial restraints. J. Mol. Biol..

[30] Kanda Y. (2013). Investigation of the freely available easy-to-use software “EZR” for medical statistics. Bone Marrow Transplant..

[31] R Core Team (2018). R: A Language and Environment for Statistical Computing.

